# Micronutrient Status in Sri Lanka: A Review

**DOI:** 10.3390/nu10111583

**Published:** 2018-10-27

**Authors:** Hansani Madushika Abeywickrama, Yu Koyama, Mieko Uchiyama, Utako Shimizu, Yuka Iwasa, Etsuko Yamada, Kazuki Ohashi, Yuta Mitobe

**Affiliations:** 1 Department of Nursing, Graduate School of Health sciences, Niigata University, 2-746, Asahimachi, Niigata 951-8518, Japan; yukmy@clg.niigata-u.ac.jp (Y.K.); uchiyama@clg.niigata-u.ac.jp (M.U.); shirabaka@clg.niigata-u.ac.jp (U.S.); iwasa@clg.niigata-u.ac.jp (Y.I.); b17a306e@mail.cc.niigata-u.ac.jp (E.Y.); 2 Department of Nursing, Saiseikai Niigata Daini Hospital, Niigata 950-1104, Japan; b16n003h@mail.cc.niigata-u.ac.jp; 3 Department of Nursing, Faculty of Medical Technology, Teikyo University, Tokyo 173-8605, Japan; b17a304j@mail.cc.niigata-u.ac.jp

**Keywords:** Micronutrients, Deficiency, Sri Lanka, Prevalence

## Abstract

Micronutrients include vitamins, minerals and, trace elements that are required in minute quantities but play a vital role in normal human growth, development and physiological functioning. Micronutrient deficiencies, also known as hidden hunger, are a global issue, with particularly high prevalence rates in developing countries. Currently, Sri Lanka is experiencing the double burden of over- and undernutrition. This review describes the micronutrient status of Sri Lanka based on results of national surveys and related articles published from 2000. The available data suggest a higher prevalence of iron, zinc, calcium, folate, and vitamin A deficiencies. The prevalence of iodine deficiency has declined gradually following the implementation of a universal salt iodization program. Iron deficiency is the most common cause of anemia and low red blood cell indices. Females are more vulnerable to micronutrient deficiencies than males. The coexistence of multiple micronutrient deficiencies and concurrent macro- and micronutrient deficiencies is common. Studies have shown an association between micronutrient deficiencies and different demographic, socioeconomic, and dietary factors. Therefore, there is a need for comprehensive studies, nutritional policies, and nationwide intervention programs in Sri Lanka to improve the micronutrient status of the population.

## 1. Introduction

Vitamins (A, B, C, D, E, and K), minerals (sodium, potassium, calcium, phosphorus, fluoride, iron, and zinc) and trace elements (iodine, copper, selenium, manganese, chromium, and molybdenum) are referred to as micronutrients because they are required in minute quantities, in contrast with macronutrients, which include energy-delivering substances such as carbohydrate, fat, and protein. Micronutrients play a vital role in normal human growth and development, physiological functioning, and maintenance of health [1]. Dietary intake of micronutrients is essential because they are not synthesized by the body. A diet inadequate in micronutrients can result in variety of negative health consequences, including decreased immunity, impaired cognitive performance, stunted growth, and increased morbidity and mortality. Determining the amount of micronutrients required is complex because it depends on many factors, including age, sex, lifestyle, hormone status, and the half-life and bioavailability of the micronutrient. Micronutrient deficiencies (MND) are progressive, and cannot be identified clinically until they are in their late stages; hence, MND are referred to as a “hidden hunger” [2].

MND are a global issue because more than two billion people are thought to be deficient, particularly in iron, zinc, iodine, vitamin A, and folate [3]. The co-existence of multiple deficiencies, as well as MND coupled with protein and/or energy malnutrition, is also common, resulting in devastating consequences. The prevalence of MND is high in developing countries, especially in Southeast Asia and Sub-Saharan Africa, and highly vulnerable groups include pregnant women, children under five years of age, and adolescents [1,3,4]. Global hidden hunger maps and indices [4] reveal alarmingly high levels of MND in India and Afghanistan in the Southeast Asia region, as well as in many countries in Sub-Saharan Africa. The study also emphasizes two aspects of the MND burden, namely the coexistence of multiple deficiencies and attributed disease burden. MND, especially zinc and vitamin A deficiency (VAD), are found to contribute to 2–12% of the total disability-adjusted life years (DALYs) in high-burden countries [4]. According to the Global Burden of Diseases (GBD) 2000 [5], iron deficiency (ID), VAD, and zinc deficiency rank 9th, 11th, and 13^th^, respectively, among 26 risk factors in terms of DALYs lost. The summary exposure values (SEV), deaths, DALYs attributable, and ranking among the leading risk factors for both sexes to ID, VAD and zinc deficiency in 2005 and 2015, as per the 2015 Global Burden of Diseases, Injuries, and Risk Factors Study, are given in Table 1 [6].

Substantial demographic and socioeconomic changes in Sri Lanka have created significant health and nutrition challenges. The nutrition transition in Sri Lanka is characterized by replacement of vegetable-based foods with animal-based foods and increased consumption of sugar, salt, and alcohol, surpassing recommended intake. In addition, the consumption of fruits, vegetables, and milk products is low [7]. Currently, Sri Lanka is experiencing the double burden of over- and undernutrition [8]. Although considerable evidence is available regarding macronutrient deficiency in Sri Lanka, there is little published information regarding MND. However, iodine status in Sri Lanka has been studied regularly through national surveys, after the initiation of a universal salt iodization (USI) program in 1995. Some small studies have reported on the prevalence of MND among different populations in Sri Lanka [9,10,11,12,13,14,15]. The aim of this review is to compile available data on MND to understand the nature and degree of MND in Sri Lanka. Data were retrieved from national nutrition and micronutrient surveys [16,17], national vitamin A survey [18], national iodine surveys [19,20,21,22] and related articles [9,10,11,12,13,14,15,23] published from 2000. The details of the studies are summarized in Table 2. All 15 studies considered in this review are cross-sectional studies.

## 2. Prevalence of MND in Sri Lanka

### 2.1. ID and ID Anemia

ID is the most prevalent MND and the main cause of anemia worldwide, with ID anemia (IDA) representing the advanced stage of ID. Although ID does not progress to anemia in all cases, anemia is often misinterpreted as an indicator of ID [24,25]. The World health organization (WHO) estimates that ID is responsible for 50% of cases of anemia in women [26], and has targeted ID in its Global Nutritional Targets 2025 in an effort to reduce anemia in women of reproductive age by 50%. The burden of ID is largely concentrated in Africa and South Asia, contributing to 75% and 65% of global mortality burden and DAILYs lost, respectively [5]. Evidence in the literature suggests that IDA is a highly prevalent nutritional problem globally, affecting almost all population groups, especially in developing countries. IDA has detrimental effects on the cognitive and physical development of children, the productivity and physical performance of adults and on pregnancy outcomes [27]. Even if it does not progress to anemia, ID may impair growth, cognition, behavior, immunity, hormone balance, performance, and work capacity [23]. Although several studies have evaluated anemia status in Sri Lanka, few have investigated ID. Because many studies in the past have misinterpreted the prevalence of anemia (based on low hemoglobin (Hb)) with that of IDA, in this review, we have only included studies that have used specific tests to diagnose ID and IDA, such as tests for serum ferritin (SF), serum iron, and serum transferrin receptor. Available data on the prevalence of ID and IDA in Sri Lanka are presented in Table 3.

Both national data [17] and data from the study of Wickramasinghe et al. (2017) [9] suggest a higher prevalence of ID and IDA during the first year of life, emphasizing the need for iron supplementation at least during the period of exclusive breastfeeding and the incorporation of iron-rich foods in complementary foods. Stored iron at birth in combination with the iron contained in breast milk is sufficient to meet the iron requirements of full-term infants during the first six months of life. Hence, low prenatal iron stores, due to preterm birth or maternal anemia, increase the risk of ID or IDA among infants [28]. In 2017, the WHO reported that there were approximately 24,500 premature births in Sri Lanka [29]. A prospective study conducted in two MOH areas in Sri Lanka reported that 82 of 694 pregnant women (12%) without maternal morbidities delivered preterm babies [30]. Senadheera et al. (2017) [15] found that 30 μg/L SF was the most appropriate cut-off value for the diagnosis of ID in pregnant women. They also found that the prevalence of both ID and IDA was highest among pregnant women aged <20 years, although the prevalence of ID was also high in pregnant women aged >40 years [15]. However, another study reported that the prevalence of IDA increased from 2.4% in the first trimester to 15.2% in the third trimester [16]. Compared with the non-pregnant state, pregnancy significantly increases iron requirements from the second trimester due to depletion of iron stores to meet the needs of the growing fetus and placental structures, as well as hemodynamic changes and losses postpartum [31].

The risk of ID and IDA during the second six months of life is particularly high due to the combined effect of increased demands, rapid development, and depleted prenatal iron stores [28]. However, according to results of the national micronutrient survey in Sri Lanka, the highest prevalence of ID within the 6–59 months age group was seen in children aged 12–23 months [17]. Increased iron demands during the growth spurt make adolescents more vulnerable to ID. Thus, Allen et al. (2017) [23] observed a higher prevalence of ID and IDA among younger adolescents (age < 16 years). In younger adolescents (age < 16 years), median transferrin receptor concentrations were significantly higher and SF and serum iron concentrations were significantly lower, suggesting higher cellular iron deficiency [23]. Low iron stores (SF level = 12–19.99 μg/L) were found among 21.3% adolescent girls and young women [14]. Therefore, subclinical ID is a significant problem that affected nearly half the populations studied [12,14,15]. The prevalence of ID did not differ significantly among adolescent girls aged 15–18.9 years and non-pregnant, non-lactating women aged 19–30 years [14]. Recent studies performed in 2014 and 2017 [17,23] have reported a lower prevalence of IDA than that reported in 2006 [12]. In their study, Allen et al. (2017) [23] used serum iron and transferrin receptor, in addition to SF, to evaluate the iron status of adolescents. The median (interquartile range) serum transferrin receptor and iron concentrations in that study were found to be 25.3 (20.91–30.58) nmol/L and 16.25 (12.19–19.93) µmol/L, respectively [23].

In terms of the sex-based prevalence of ID and IDA, a national survey reported significantly higher prevalence among male than female children [17], whereas no such sex-based differences were found by Hettiarachchi and Liyanage [11]. In contrast, a significantly higher prevalence of ID and IDA was observed among adolescent females than males [12,23], which, as noted above, is possibly related to the occurrence of menarche in females. The median age of menarche and attaining an adult state in Sri Lanka has been found to be 11.2 and 15.5 years old, respectively [32]. Further, female adolescents reported significantly lower SF, transferrin receptor, and iron values compared to their counterparts (*p* value < 0.001) [23]. Hettiarachchi et al. (2006) reported improved mean SF levels with age among adolescent males and rapid decline in SF among adolescent girls with the onset of menstruation [12]. The prevalence of ID and IDA according to sex is summarized in Table 4.

### 2.2. Iodine Deficiency

Iodine deficiency, considered the most easily preventable MND, is associated with many adverse effects on growth and development that are collectively known as iodine deficiency disorders (IDD). Classic signs of iodine deficiency are thyroid enlargement or goiter, with other IDD including abortion, stillbirths, congenital anomalies, and secondary effects of hypothyroidism [33]. Iodine deficiency was identified as a major health problem in Sri Lanka in the late 1940s. Earlier studies found a higher incidence of goiter particularly in the wet zone of Sri Lanka, which was subsequently termed the “goiter belt” [34,35]. The major etiological factor for endemic goiter was found to be a lack of iodine in the food and water. The national IDD survey conducted in 1986 found a total prevalence of goiter among school children of 18.2% [35]. Consequently, the USI program was implemented in Sri Lanka in 1995 with the goal of maintaining median urinary iodine (UI) concentrations in the Sri Lankan population of 100–200 μg/L, reducing the goiter rate to <5% in school children, and ensuring that >90% of households use 90% iodized salt. Hence, legislation was adopted to maintain the iodine content in salt at 50 and 25 ppm at the manufacturing and consumer levels, respectively [19]. 

During 2000–2001, Medical Research Institute in Sri Lanka investigated the effectiveness of the USI program at the national level using a representative sample of 8–10-year-old school children. In that study, median UI concentrations were above 100 μg/L in all provinces except Uva province [22]. Even though UI concentrations in the North Central Province (NCP) were more than adequate, the total goiter rate (TGR) was similar in Uva and NCP provinces. Further, there were wide variations in iodine content between salt brands and within a given brand, high goiter rates were seen with both insufficient (<20 μg/L) and excessive (>300 μg/L) UI concentrations [22]. Thus, the authors postulated that other goitrogens, such as selenium deficiency, could be contributing to the occurrence of goiter [22]. However, a second national IDD survey conducted in 2005 [21] confirmed that the standards set by a Joint committee from the WHO, UNICEF, and the International Council for Control of Iodine Deficiency Disorders for the elimination of IDD had been achieved. These findings led to a revision of the salt iodization legislation, now requiring maintenance of iodine in salt at levels of 15 and <30 ppm at the household and production levels, respectively [19]. The third and fourth national iodine surveys were conducted in 2010 [20] and 2016 [19], and the findings of all four surveys are summarized in Table 5. 

The current TGR in Sri Lanka is well below the public health cut-off of 5%, and the median UI concentrations in all nationwide studies were above the acceptable level of 100 μg/L [19,20,21,22], indicating optimal iodine status and thyroid health among school-age children. However, median UI concentrations were above 200 μg/L (more than adequate) in the NCP and Northern provinces of Sri Lanka. Chronic excess iodine intake in populations with iodine deficiency may lead to thyroid dysfunction and iodine-induced hypothyroidism, as has been reported in other countries during the introductory phase of USI programs [33]. Another study also reported higher prevalence of anti-thyroglobulin (TgAb) antibody among adolescent girls and emphasized the need for continuous monitoring of adequacy and risks of iodization [36]. All nationwide surveys reported the highest goiter prevalence rates and lowest UI concentrations in females [19,20,21,22]. In addition to national surveys, a few other studies measured and reported iodine deficiency among different population groups [11,16,36,37], and their findings are summarized in Table 6. 

### 2.3. Other Mineral Deficiencies

Zinc is an essential trace mineral for cellular metabolism, immune function, linear growth, and neurobehavioral development. Its role in cell division, enzyme function, and protein and DNA synthesis, zinc is a critical determinant of normal pregnancy outcomes and child growth. Zinc deficiency increases the incidence, morbidity, and mortality of diarrhea, acute respiratory infections, and malaria [4,38]. Unlike iron, there is no long-term storage system for zinc in the human body, and therefore consistent dietary intake is essential. Major dietary sources of absorbable zinc are animal-based foods and seafoods [4]. Thus, zinc deficiency may be common in populations consuming a cereal-based diet with low levels of food from animal sources. The WHO has estimated that, annually, 1.4% of deaths (0.8 million) worldwide are attributable to zinc deficiency [39]. According to 2012 estimations [38], 17.3% of the global population and 30% of the population in South Asia is at risk of inadequate intake of zinc. 

A lack of nationally representative data hampers validation of the magnitude of other mineral deficiencies in Sri Lanka. Available data on the prevalence of zinc, calcium, and copper deficiencies are presented in Table 7. Earlier studies report higher prevalence (>50%) of zinc deficiency among preschool aged children and adolescents [10,11,12], whereas, in the national survey [17], the prevalence of zinc deficiency was much lower than the 20% set by the International Zinc Nutrition Consultation Group [40] as an indicator of the need for national intervention programs. This may be due to changes in dietary patterns characterized by an increase in the consumption of animal-based foods, as well as increases in nutrition-related knowledge and/or the effectiveness of intervention programs. In most studies [10,11,17], there were no significant differences in the prevalence of zinc deficiency or in mean zinc concentrations between males and females; however, Hettiarachchi et al. (2006) [12] found significantly higher age-adjusted mean zinc concentrations levels among adolescent boys than girls (*p* < 0.001). 

In the national survey [17], a higher prevalence of calcium deficiency was found among children aged 6–59 months, with nearly half the study population being affected. In contrast, in the Galle district, the prevalence of calcium deficiency among preschool-aged children was less than 10% [11]. The difference between findings in Jayatissa et al. [17] and Hettiarachchi and Liyanage [11] vs. Marasinghe et al. [10] may be due to the use of serum calcium vs. serum PTH. Calcium homeostasis is tightly regulated by a complex mechanism and extracellular fluid calcium concentration and body calcium content are largely independent variables. Therefore, serum calcium concentration is considered a poor indicator of calcium status [41]. There were no significant differences in the prevalence of calcium deficiency between male and female children [11,17]. The only study on copper used serum ceruloplasmin as an indicator of copper concentrations and found no significant difference in mean and low ceruloplasmin concentrations according to age and sex [11]. A significant proportion of females from areas with a low, medium, or high prevalence of IDD was found to be selenium deficient (24%, 24%, and 40%, respectively) [42]. Relation of selenium deficiency with chronic kidney disease of uncertain etiology (CKDu); a disease predominant among male farmers in the dry zone of Sri Lanka has also been implicated [43]. Recent studies have reported that a substantial population of the Sri Lankan population is selenium deficient, regardless of sex and age [44,45].

### 2.4. Vitamin Deficiencies

In addition to being important for vision health, vitamin A has roles in reproduction, cellular growth and differentiation, immune function, and maintenance of epithelial cell integrity. Hence, VAD can have several negative health consequences, including childhood blindness, xeropthalmia, impaired growth and development, and increased rates of infections, morbidity, and mortality [46]. According to WHO estimates [47], 250 million preschool children worldwide are vitamin A deficient and, of these, between 2.5 × 10^5^ and 5 × 10^5^ become blind annually. VAD is more prominent among young children and pregnant women in developing countries. Available data on the prevalence of vitamin A, D, B_12,_ and folate deficiencies in Sri Lanka are summarized in Table 8.

The 2006 national survey of vitamin A status in Sri Lanka [18] recognized VAD as a significant public health problem, with a prevalence of 29.6% among children aged 1–5 years and 2.3% of children in the entire sample having severe deficiency (serum retinol < 10 μg/L). According to the recommendations of the International vitamin A consultation group [48], when 15% of children aged 1–5 years in a given population have serum retinol concentrations < 20 μg/L, the community has a VAD of public health significance. The highest prevalence of overall and severe VAD was observed in children aged 6–11 and 24–35 months, respectively [18]; however, clinical examinations revealed that none of the children had Bitot’s spots or night blindness. The prevalence of VAD was higher among girls than boys for both moderate and severe deficiency [18]. In another study in an urban MOH area, a much higher prevalence of VAD was observed, with similar prevalence rates between boys and girls [10]. Although these results suggest that VAD is an important public health concern, most recent studies have reported a lower prevalence VAD [9,11,16]. Pathmeswaran et al. (2005) investigated the prevalence of Bitot’s spots among school-aged children and found that only a small proportion (0.3%) had clinically obvious VAD [37]. In another study, 72% of the sample of preschool-aged children in Galle district was found to be vulnerable to VAD, with marginal vitamin A concentrations (0.70–1.04 mmol/L), but only 5% of the sample was vitamin A deficient [11]. This indicates the higher prevalence of a suboptimal vitamin A status rather than actual deficiency. 

Even though Sri Lanka is a tropical country with sufficient sunlight throughout the year, Hettiarachchi and Liyanage (2012) found vitamin D deficiency (VDD) in over one-quarter of their sample, although they did not find anyone with severe hypovitaminosis D (serum 25-hydroxyvitamin D < 12.5 nmol/L) [11]. Conversely, Marasinghe et al. (2015) [10] reported a low prevalence (5%) of VDD among preschool children, although 29% had vitamin D insufficiency (serum 25-hydroxyvitamin D = 10–20 ng/mL). These authors also observed a significant inverse correlation between serum 25-hydroxyvitamin D and serum PTH, which was used as a surrogate marker of serum ionic calcium [10]. These findings suggest that serum calcium levels increase with increasing vitamin D levels. There were no significant differences in mean serum 25-hydroxyvitamin D concentrations according to sex in either study (*p* > 0.05) [10,11]. Thus far, only two studies have investigated serum Vitamin B_12_ levels [13,14], with low serum Vitamin B_12_ (<150 pg/mL) levels being found in only 10 of 613 [13] and 2 of 600 subjects [14]. However, higher prevalence rates of folate deficiency were observed [11,12,13,14]. Thoradeniya et al. (2006) did not find any significant difference in the prevalence of folate deficiency between adolescent girls (45.1%) and women of childbearing age (42.1%) [14]. There were no significant differences in folate deficiency according to sex and age groups of children and adolescents in Galle district [11,12]. 

### 2.5. Coexistence of Multiple MND

The simultaneous occurrence of multiple MND is more common than single MND occurring in isolation. Available data suggest a higher prevalence of multiple MND among children and adolescents in Sri Lanka; however, it is difficult to estimate the gravity of the situation because of a lack of data. Of a cohort of preschool children in Galle district, 62% were found to have multiple MND [11]. In the sample, 38.3%, 17.7%, and 6% of children had two, three, or four or more MND simultaneously, and only 7.3% did not have any type of MND studied [11]. Another study in Galle district also highlighted the widespread nature of coexisting MND among adolescent school children, with iron-deficient subjects having a 1.8- and 1.7-fold risk of being folate and zinc deficient, respectively [12]. Similarly, zinc-deficient subjects had a 1.3-fold higher risk of being iron deficient and a 1.2-fold higher risk of being folate deficient. In the same study, 20.5% and 24% of zinc-deficient children were also deficient in iron and folate, respectively, whereas 16% of subjects had both iron and folate deficiency [12]. One of the major findings of the study of Marasinghe et al. (2015) was that none of the children studied was free from any MND, whereas 92% of the children had two or more coexisting MNDs. Nearly half of the subjects in that study were suffering from two MND simultaneously, whereas approximately 10% had four coexisting MND [10]. In another study, there was a significant difference in the incidence of low serum folate plus low SF between anemic and non-anemic subjects (34% vs. 79% respectively; *p* < 0.001) [14]. 

## 3. Factors Associated with MND

### 3.1. Demographic and Socioeconomic Factors

Allen et al. (2017) identified female sex as the greatest risk factor for ID, IDA, and cellular iron deficiency (low SF and raised serum transferrin receptor), with adolescent girls having a threefold greater risk than boys for all three stages of low iron status. This finding is likely due to menstrual loss not compensated for by dietary intake [23]. In contrast, the national survey on MND reported a higher prevalence of ID, IDA, and zinc deficiency in boys under five years of age compared with girls, even though there was no sex difference with regard to calcium deficiency [17]. The proportion of girls under five years of age with either severe or moderate VAD was greater than the proportion of boys, although the difference did not reach statistical significance difference [18]. National iodine surveys have also reported a higher prevalence of iodine deficiency among females than males [19,20,21,22].

The highest prevalence of both ID and IDA was found among Tamils, followed by Sinhalese and Muslims [23]. In addition to dietary patterns, the poor iron status of Tamils is likely due to socioeconomic disadvantage as a result of long lasted war and working in the tea estate sector. Further, ID was most common in the northern part of Sri Lanka, a region that was affected by war for decades and where the majority of people are Tamils [23]. Living at high altitudes significantly reduces the risk of ID [23], whereas the risk for iodine deficiency was higher at higher altitudes [19,20,21,22]. In addition, UI concentrations were consistently higher in the NCP and Northern provinces, which may lead to iodine-induced hyperthyroidism [19,20,21,22]. Selenium deficiency in Sri Lanka likely contributes to goiter in the wet zone [42] and to CKDu in the dry zone [43]. The prevalence of ID, IDA, VAD, and iodine deficiency among pregnant women was highest for those working in the tea estate sector, whereas lowest values for ID and IDA prevalence were found among those working in the rural sector [16]. 

The prevalence of ID decreases significantly with an increase in the number of household members, even though the prevalence of ID is higher in households in the lowest income and wealth quintiles [17]. Conversely, zinc and calcium deficiencies do not show any consistent pattern with household income, and even though there is a significant decline in zinc deficiency with increasing wealth quintile, there is a marginal increase in the prevalence of zinc deficiency in the highest wealth quintile [17]. There are also significant differences in mean folic acid concentrations among income groups [14]. Education level affects the health status of people globally, and the national vitamin A survey in Sri Lanka found that a higher level of education of mothers associated with a low prevalence of VAD [18]. Jayatissa et al. (2014) observed significant differences in the prevalence of ID and zinc and calcium deficiencies according to mother’s education level, even though there were no clear patterns. The prevalence of zinc and calcium deficiency was higher among children whose mothers had been educated up to the primary and secondary levels, respectively [17]. 

Employment has been identified as a major factor that increases the vulnerability of adolescent girls (aged 15–19 years) to MND, with those who are working having a nearly two-fold higher risk of developing ID (*p* = 0.009) and folate deficiency (*p* = 0.006) and a 2.1-fold higher risk of developing zinc deficiency (*p* < 0.001) [13]. Dropping out of school at <14 years of age was found to increase the risk for ID by 2.1-fold (*p* = 0.01) compared with dropping out of school at >14 years of age [13]. The relationship between a father’s employment and the prevalence of ID, zinc, and calcium deficiency was assessed in the national survey of MND [17]. Although there was not much variation in the prevalence of ID in children according to father’s employment, the lowest and highest prevalence of both zinc and calcium deficiency was seen in children whose fathers belonged to the “managerial” and “unskilled laborers” categories, respectively [17]. 

### 3.2. Nutritional Status and Anthropometric Factors

Marasinghe et al. (2015) [10] found a significant relationship between vitamin D status and the height and weight of preschool children. In that study, a significant decrease was seen in serum 25-hydroxyvitamin D levels with increasing levels of stunting (moderate to severe), which is in agreement with the role of vitamin D in the skeletal growth of children. Although the correlation was not significant, zinc-deficient children had lower *Z*-scores for weight for height, weight for age, and height for age [10]. Similarly, mean serum vitamin A concentrations decreased with increasing levels of undernutrition, with severely stunted children having the lowest mean serum vitamin A concentration (16 μg/dL). However, there was no association between nutritional status and VAD among children under five years of age [10]. Interestingly, one study reported that the prevalence of VAD was highest among women with normal nutritional status and lowest among obese women [18]. In another study [49], in a cohort of adolescent school children, body mass index (BMI) was positively correlated with dietary iron intake (Spearman *p* = 0.30, *p* < 0.01), whereas zinc intake was negatively correlated with BMI *Z*-score (*p*= −0.06, *p* = 0.04). In the same study, a negative correlation was found between height for age and iron intake (*p* = −0.14, *p* < 0.01), whereas a positive correlation was found between stunting and zinc intake (*p*= 0.06, *p* = 0.05) [49].

Although there was no significant difference in micronutrient status between normal and undernourished infants [9], the prevalence of ID was higher among infants with normal nutritional status than in the undernourished group. The authors of that study suggested that this may be associated with the initiation of complementary feeding before six months of age for 73% of infants without any medical advice, which leads to the intake of foods rich in calories but deficient in micronutrients [9]. In a national survey [17], the prevalence of ID, IDA, zinc, and calcium deficiency was higher among overweight children, but there were no significant differences in ID and IDA among children with normal, stunting, wasting, or underweight nutritional status. However, the prevalence of zinc deficiency among children with normal and stunted nutritional status was similar and lower than that in the wasted and underweight groups. The prevalence of calcium deficiency among stunted and underweight groups was approximately 50% [17]. 

### 3.3. Dietary Factors

The consumption of vitamin A-rich foods by children under five years of age was assessed in the national survey of vitamin A status, and it was found that average consumption was lower than acceptable levels [18]; based on these findings, the authors identified VAD as a public health concern with regard to the consumption of vitamin A-rich foods [18]. Among a cohort of preschool children in Galle district, folate intake was found to be less than the recommended dietary allowance (RDA), whereas zinc intake was only half the RDA [50]. That study also reported higher iron intake among girls, which was double the RDA [50]. Significant differences (*p* = 0.01) in dietary zinc intake have been observed between adolescent boys and girls, as well as in iron intake between pre- and postmenarcheal girls [49], with the authors of that study concluding that there was inadequate dietary intake of energy, protein, and micronutrients among adolescent school children [49]. Another study reported significantly lower dietary folate intake among subjects with low serum folate compared with their counterparts (*p* < 0.001), with folate intake significantly associated with the education level, monthly income, and number of household members [14]. In a national micronutrient survey investigating the association of iron and zinc deficiency and the intake of eight different food groups (and the mean number of days of consumption), there was no significant association between food intake and iron or zinc deficiency [17]. However, a statistically significant association was found between the frequent consumption of dairy products and the occurrence of ID. In addition, a significantly higher prevalence of iron and zinc deficiency has been reported among children who consume bottled water [17]. Low selenium intake among the Sri Lankan population has been reported previously [42], and Sri Lanka is ranked among the top three South Asian countries in terms of inadequate intake of vitamin A, vitamin B_12_, calcium, and riboflavin [51].

### 3.4. Anemia

Low iron and folate levels have been recognized as significant predictors of nutritional anemia, with the risk of anemia in subjects with depleted iron stores (SF < 12 mg/L) and low folate status (serum folic acid concentration < 3 ng/mL) being 6- and 2.3-fold greater, respectively, than in than in iron replete and serum folic acid concentration >3 ng/mL subjects [14]. Interestingly, subjects with concurrent iron and folate deficiency did not have a significantly higher risk of anemia than subjects who were deficient in only one of these parameters. However, prevalence of vitamin B_12_ deficiency among adolescent girls and young women was very low, so it is unlikely to have a role in anemia [14]. Both preschool and adolescent girls with anemia in Galle district were found to be more vulnerable to MND than boys [11,12]. Table 9 summarizes the available data on prevalence and risk of anemic children having MND. Marasinghe et al. (2015) observed significant positive correlations between Hb and zinc, vitamin A, and vitamin D [10]. Conversely, Hb levels have been reported to be positively correlated with SF and serum retinol, whereas a negative correlation has been reported with Hb and vitamin D [11].

In a recent study, ID was recognized as the most common cause of low values of red blood cell indices, such as mean corpuscular volume < 80 fL and/or mean corpuscular hemoglobin < 27 pg, in Sri Lanka, followed by α thalassemia [52]. This complicates the use of red blood cell indices in the screening for thalassemia carriers, and emphasizes the need to identify of the exact cause of the low values of red blood cell indices prior to administration of iron supplements [52]. In the national survey on MND, 12.8% of anemic children were found to have hemoglobinopathies and 8% of those with a hemoglobinopathy also had ID [17]. In another study, ID did not account for anemia among 16.4% of adolescent boys and 9.1% of girls [23]. However, the percentage of anemic children and adolescents with ID in Sri Lanka [9,12,17] was approximately 50%, which is on par with WHO estimates. This indicates the contribution of other possible causes in addition to ID to the high prevalence of anemia in Sri Lanka, such as infections, inflammation, other MND (folate, vitamin B_12_, and vitamin A), and hemoglobinopathies including α- and β-thalassemia, and HbE [53]. A recent study found that anemia was common (27%) among adolescents with hemoglobinopathies [52]. Furthermore, the study reported that the frequency of low iron stores (<15 ng/mL) in adolescents with a normal Hb genotype or hemoglobinopathy was 37% and 30.5%, respectively [52]. 

### 3.5. Other Factors

In 2001, a vitamin A supplementation program was initiated in Sri Lanka that provided children with four doses of vitamin A (100,000 IU) at 9 and 18 months of age and then again at five and nine years of age, as well as a vitamin A megadose (200,000 IU) to mothers within four weeks after delivery. The national survey in 2006 revealed that 66% of children under five years of age had received vitamin A supplementation at least once, and that the prevalence of VAD immediately and six months after supplementation was 23.9% and 34%, respectively [18]. Based on these findings, it was recommended that vitamin A doses be administered at six-monthly intervals from six months to five years of age, providing 10 megadoses of vitamin A; this recommendation has been implemented [18]. Another study reported similar findings, whereby children supplemented with a vitamin A megadose had significantly higher serum vitamin A concentrations than children who did not receive the supplement [54]. The authors of that study found that the time of supplementation was the main predictor of serum vitamin A concentrations, which decreased gradually after administration [54]. Furthermore, the prevalence of iron and zinc deficiencies was significantly lower in children who received multiple micronutrient supplements, and the administration of deworming tablets also had a positive effect on both deficiencies [17].

Even though serum retinol concentrations were higher in breastfed than non-breastfed children between 6 and 23 months of age [18], no association was found between breastfeeding and ID and zinc deficiency [9,17]. Thoradeniya et al. (2006) found that mean serum folic acid levels were significantly lower among parous than nulliparous women [14], whereas in another study median UI concentrations decreased with increasing gestational weeks [20]. Approximately 60% of LBW infants and one-third of normal birth weight infants were found to be iron deficient at six months of age despite iron supplementation [9]. Although the difference did not reach statistical significance, vitamin A levels tended to be lower in the low compared with normal birth weight infants [9]. In another study [18], the prevalence of respiratory tract infections was significantly higher in children under five years of age with than without VAD. Furthermore, children with diarrhea had severe VAD, whereas the prevalence of VAD was similar among children with or without diarrhea [18]. 

## 4. Conclusions

Determining the prevalence of MND among different population groups in Sri Lanka is hampered by the lack of nationally representative data. However, the available data suggest a higher prevalence of iron, zinc, calcium, folate, and vitamin A deficiencies. The prevalence of iodine deficiency has declined gradually after the implementation of the USI program. Limited data are available for other MND, including calcium, selenium, copper, vitamin D, and vitamin B_12_, possibly due to the lack of specific and sensitive indicators, economic considerations, and/or complicated assessment procedures. ID is the most common cause of anemia and low values of red blood cell indices. Females are more vulnerable to MND than males. One of the key issues is the coexistence of multiple MND, affecting more than 50% of children and adolescents in Sri Lanka. A higher prevalence of concurrent MND emphasizes the importance of focusing on several micronutrients, rather than just one, and their interactions during screening for and treatment of MND. In addition, macro- and micronutrient deficiencies occur simultaneously. Studies have shown the association of MND with different socioeconomic and dietary factors, nutritional status, and anemia at different levels. The focus of many studies has been on children and adolescents, and there is a significant gap in the literature with regard to the micronutrient status of other vulnerable groups, such as pregnant and lactating women and the elderly.

Supplementation and food fortification are the most commonly used strategies to alleviate MND in Sri Lanka. In particular, salt iodization and vitamin A supplementation have proven to be successful [18,19,20,21,22]. Rice and rice flour have been suggested as potential vehicles for fortification due to their high consumption, availability, and affordability [55,56]. An ideal approach to reducing MND is to improve the diet quality of the population, because this increases the simultaneous intake of many nutrients [57]. Low dietary diversity has been reported among children and the elderly [58,59], but some studies have reported the consumption of a diverse diet, albeit in low quantities [60,61]. Therefore, a broader and positive understanding of diet and nutrition in the population must be built through advocacy and communication. In addition, researchers and authorities must pay attention to biofortification, which is a feasible strategy for the reduction of MND. A study that prioritized countries for biofortification interventions identified Sri Lanka as a top priority country for the biofortification of rice with zinc [62]. Hence, comprehensive studies on micronutrient status, including both epidemiological and interventional studies, are an important prerequisite to ensure that adequate data are available to enable decisions to be made, new policies to be developed and new initiatives to be implemented, contributing to the improved health status of the Sri Lankan population. 

## Figures and Tables

**Table 1:** Global age-standardized SEV, all-age deaths, DALYs attributable and ranks allocated to ID, VAD and zinc deficiency in 2005 and 2015 [6].

MND	SEV (%)				Attributable Global Deaths (Per 1000 Persons)		Attributable DALYs Lost (Per 1000 Persons)		Ranking among Leading Risk Factors	
	2005		2015		2005	2015	2005	2015	2005	2015
	Male	Female	Male	Female						
ID	NA	17.5	NA	16.5	87	84	55 120	52 870	17	16
VAD	32.4	29.2	28.4	25.8	191	83	16 864	7 611	26	39
Zinc deficiency	16.8	16.8	15.6	15.6	93	55	8 162	4 967	36	40

The summary exposure value (SEV) is the risk weighted prevalence of an exposure. The scale for SEV ranges from 0% to 100%, where 0% reflects no exposure and 100% reflects maximum possible risk in a population. NA, Relevant data not available. MND, micronutrient deficiency; ID, iron deficiency; VAD, vitamin A deficiency; DALYs, disability-adjusted life years.

**Table 2:** Characteristics of the studies included in this review.

Author and Year	Study Area	Population Studied	Sample Size	Micronutrients Studied
Jayatissa, Fernando and De Silva, 2017 ^A^ [16]	25 districts	Pregnant women	7500	Iron, iodine, Vitamin A
Jayatissa et al., 2014 ^A^ [17]	25 districts	Children aged 6–59 months	7500	Iron, Zinc, Calcium
Jayatissa, Fernando and Herath, 2016 ^B^ [19]	9 provinces	School children aged 6–12 years	8624	Iodine
Jayatissa and Gunathilaka, 2012 ^B^ [20]	9 provinces	School children aged 6–10 years	8060	Iodine
		Pregnant women	587	
Jayatissa and Gunathilaka, 2006 ^B^ [21]	9 provinces	School children aged 6–9 years	1900	Iodine
Jayatissa, Gunathilaka and Fernando, 2005 ^B^ [22]	9 provinces	School children 8–10 years	7076	Iodine
Jayathissa and Gunathilaka, 2006 ^C^ [18]	20 districts	Children 6–60 months	900	Vitamin A
Allen et al., 2017 [23]	25 districts	Secondary school children 11–19 years	7526	Iron
Wickramasinghe et al., 2017 [9]	Colombo MC area	Infants aged 6–6.5 months	96	Iron, Vitamin A
Marasinghe et al., 2015 [10]	Ragama MOH area	Pre-school children aged 2–5 years	340	Zinc, Calcium, Vitamin A, Vitamin D
Hettiarachchi and Liyanage, 2012 [11]	Galle district	Pre-school children aged 3–5 years	248	Iron, Zinc, Calcium, Copper, Iodine, Vitamin A, Vitamin D, Folate
Hettiarachchi et al., 2006 [12]	Galle district	Secondary school children 12–16 years	945	Iron, Zinc, Folate
de Lanerolle-Dias et al., 2012 [13]	Western Province	Girls who dropped out of school (age 15–19 years)	613	Iron, Zinc, Folate, Vitamin B_12_
Thoradeniya et al., 2006 [14]	Colombo MC area	Adolescent girls (age 15–18.9 years) and non-pregnant, non-lactating young women (age 19–30 years)	600	Iron, Folate, Vitamin B_12_
Senadheera et al., 2017 [15]	Antenatal clinic at Teaching hospital, Mahamodara, Galle	Pregnant women between 12 and 20 weeks gestation	350	Iron

^A^ National nutrition and micronutrient survey. ^B^ National iodine survey. ^C^ National vitamin A survey. MC, municipal council; MOH (medical officer of health units) are responsible for preventive and promotional healthcare in a defined area in Sri Lanka. Provinces and districts are the first and second level of administrative divisions in Sri Lanka, respectively, and Sri Lanka is divided into nine provinces and 25 districts.

**Table 3:** Prevalence of ID and IDA in Sri Lanka.

Author and Year	Population Studied	Mean SF Level (µg/L)	ID Prevalence (%)	IDA Prevalence (%)
Jayatissa et al., 2014 ^A^ [17]	Children aged 6–59 months	NA	33.6 ^a^	7.4 ^e^
Jayatissa, Fernando and De Silva, 2017 ^A^ [16]	Pregnant women	NA	21.8 ^b^	10.8 ^f^
Wickramasinghe et al., 2017 [9]	Infants aged 6–6.5 months	15.5	37.2 ^a^	NA
Allen et al., 2017 [23]	Secondary school children aged 11–19 years	NA	19.2 ^b^	3.9 ^g^
Hettiarachchi et al., 2006 [12]	Secondary school children aged 12–16 years	M 35.03 F 26.62 ^B^	14.7 ^a^ 24.5 ^d^	33.9 ^h^
de Lanerolle-Dias et al., 2012 [13]	Girls who dropped out of school (age 15–19 years)	NA	29.4 ^c^	NA
Thoradeniya et al., 2006 [14]	Adolescent girls (age 15–18.9 years) and non-pregnant, non-lactating young women (age 19–30 years)	19.7	25·3 ^a^	NA
Senadheera et al., 2017 [15]	Pregnant women between 12 and 20 weeks gestation	47.7	3.7 ^a^ 36.9 ^d^	NA

^A^ National survey. ^B^ Geometric mean. NA, Relevant data not available. M, Male; F, Female; SF, Serum ferritin; ID, Iron deficiency; IDA, Iron deficiency anemia. ID was diagnosed on the basis of SF concentrations as follows: (a) <12 μg/dL; (b) <15 μg/dL; (c) *<*20 μg/L; and (d) <30 μg/L. IDA was diagnosed on the basis of SF and hemoglobin (Hb) concentrations as follows: (e) SF < 12 μg/dL with low Hb <11 g/dL; (f) SF < 15 μg/dL with low Hb <11 g/dL; (g) SF *<* 15 ng/mL, increased transferrin receptor >28.1 nmol/L and low Hb <11.5.0 g/dL in children aged <12 years, Hb < 12.0 g/dL in females aged ≥12 years and in males aged 12–14 years and Hb < 13.0 g/dL in males aged ≥ 15 years; and (h) SF < 30 μg/L and Hb < 120 g/L.

**Table 4:** Prevalence of ID and IDA according to sex.

Author and Year	Population Studied	Sample Size	Deficiency	Prevalence (%)		* *p* Value
				Male	Female	
Jayatissa et al., 2014 (National survey) [17]	Children aged 6–59 months	M 2902 F 2864	ID ^a^	36.1	31.0	0.00 ^A^
			IDA ^b^	8.5	6.2	0.01 ^A^
Hettiarachchi and Liyanage, 2012 [11]	Pre-school children aged 3–5 years	M 122 F 126	ID ^c^	37	33	NA
			IDA ^d^	2	5	0.59 ^B^
Allen et al., 2017 [23]	Secondary school children aged 11–19 years	M 2876 F 2885	ID ^e^	11.2	27.1	<0.001 ^C^
		M 2785 F 2794	IDA ^f^	1.0	4.6	<0.001 ^C^
Hettiarachchi et al., 2006 [12]	Secondary school children aged 12–16 years	M 327 F 555	ID ^c^	7	11.2	<0.001 ^B^
			IDA ^g^	26.6	43	<0.001 ^A^

* *p* values < 0.05 considered significant. NA, not available. M, Male; F, Female; ID, Iron deficiency; IDA, Iron deficiency anemia. ^A^ Two-sample *t*-test, ^B^ Chi-square test, ^C^ *p* value for the variable in multiple regression analysis. Iron deficiency (ID) was diagnosed based on serum ferritin (SF) concentrations as follows: (a) <12 μg/dL; (c) < 30 µg/L; and (e) *<*15 ng/mL. IDA was diagnosed based on SF and hemoglobin (Hb) concentrations as follows: (b) SF < 12 µg/dL with low Hb < 11 g/dL; (d) SF < 12 µg/L with low Hb <110 g/L; (f) SF *<*15 ng/mL, increased transferrin receptor >28.1 nmol/L, and low Hb <11.5.0 g/dL in children aged < 12 years, Hb < 12.0 g/dL in females aged ≥ 12 years and in males aged 12–14 years, and Hb < 13.0 g/dL in males aged ≥ 15 years; and (g) SF < 30 μg/L and Hb < 120 g/L.

**Table 5:** Findings of national iodine surveys in 2000 [22], 2005 [21], 2010 [20] and 2016 [19].

Variables	Year of Survey			
	2000	2005	2010	2016
Total goiter rate (%)	20.9	3.8	4.4	1.8
Median UI concentration (μg/L)	145.3	154.4	163.4	232.5
Percent of subjects with:				
Adequate UI levels (100–199.9 μg/L)	35.4	34.7	37.5	NA
More than adequate UI levels (200–299.9 μg/L)	17.8	18.7	22.2	NA
Excessive UI levels (>300 μg/L)	16.3	16.8	14.9	29.5
Iodine deficiency (<100 μg/L)	30.6	29.9	25.5	NA
Severe iodine deficiency (<20 μg/L)	1.4	0.1	1.4	NA
Mean iodine content in salt at household level (ppm)	NA	28	21.2	21.2
Percent of households with access to adequate iodine in salt	49.5	91.2	51.2	78.5

NA, not available. UI, Urinary iodine; ppm, parts per million.

**Table 6:** Findings related to Iodine deficiency among different population groups.

Author and Year	Study Area	Sample Size	Population Studied	Criteria	Findings
Jayatissa, Fernando and De Silva, 2017 [16]	25 districts	980	Pregnant women	UI concentration	Median UI concentration—157.9 μg/dL
					52.2% had UI concentration >150 μg/dL
					10.1% had UI concentration > 50 μg/dL
Jayatissa and Gunathilaka, 2012 [20]	9 provinces	587	Pregnant women	UI concentration	Median UI concentration—113.1 μg/L
					62.5% were iodine deficient (UI concentration < 150 μg/L)
					14.5% had above requirement (250–499 μg/L) UI concentration
					1.9% had excessive (>500 μg/L) UI concentration
Hettiarachchi and Liyanage, 2012 [11]	Galle district	248 (M 122 F 126)	Pre-school children aged 3–5 years	Serum free T4	Median free T4 concentration for M—14.83 pmol/L F—15.08 pmol/L
					None of the children had low thyroxin levels (Serum free T4 < 10.30 pmol/L)
Premawardhana et al., 2000 [36]	-	367	School girls aged 11–16 years	Ultrasound thyroid volume, Free T4, Free T3, TSH, UI concentrations, TgAb	Normal median thyroid volume, UI concentrations observed.
					Free T4 and free T3 were normal in all subjects.
					TSH was elevated in four subjects
					Higher prevalence of TgAb, which reflect
					The high prevalence of TgAb, which reflects excessive iodination of Tg resulting in increased immunogenicity.
Pathmeswaran et al., 2005 [37]	9 provinces	2528 (M 1177 F 1351)	Grade 5 school children	Visible or palpable enlargement of thyroid gland	Goiter prevalence rate- 3%
					Prevalence rates were significantly higher among girls than boys *

M. Male; F. Female; UI. Urinary iodine; T4. thyroxine; T3, tri-iodothyronine; TSH, Thyrotrophin; TgAb, anti-thyroglobulin antibody. * Chi-square = 20.3, *p* < 0.001.

**Table 7:** Prevalence of zinc, calcium and copper deficiencies in Sri Lanka.

Author and year	Area	Population	Prevalence (%)		
			Zinc Deficiency	Calcium Deficiency	Copper Deficiency
Jayatissa et al., 2014 ^A^ [17]	25 districts	Children aged 6–59 months	5.1 ^a^	47.6 ^f^	NA
Marasinghe et al., 2015 [10]	Ragama MOH area	Pre-school children aged 2–5 years	66.7 ^b^	12.06 ^g^	NA
de Lanerolle-Dias et al., 2012 [13]	Western Province	Girls who dropped out of school (age 15–19 years)	28.8 ^c^	NA	NA
Hettiarachchi and Liyanage, 2012 [11]	Galle district	Pre-school children aged 3–5 years	~50 ^d^	M 8, F 6 ^h^	M 7, F 1 ^i^
Hettiarachchi et al., 2006 [12]	Galle district	Secondary school children aged 12–16 years	55.7 ^e^	NA	NA

NA, not available. M, Male; F, Female; MOH, Medical officer of health. ^A^ National survey. Zinc deficiency was diagnosed based on serum zinc concentrations as follows: (a) <65 μg/dL in the morning and <57 μg/dL in the afternoon; (b) <9.9 μmol/L; (c) <66 μg/dL; (d) <9.945 mmol/L; and (e) <9.95 μmol/L. The criteria used to diagnose calcium deficiency were as follows: (f) serum calcium 8.4 mg/dL; (g) serum parathyroid hormone (used as a surrogate marker of serum ionized calcium) > 65 pg/mL; and (h) serum calcium <1.20 mmol/L. (i) Copper deficiency was diagnosed based on serum caeruloplasmin (used as a surrogate marker for copper levels) <240 mg/L.

**Table 8:** Prevalence of vitamin deficiencies in Sri Lanka.

Author and year	Area	Population	Prevalence (%)			
			Vitamin A Deficiency	Vitamin D Deficiency	Vitamin B_12_ Deficiency	Folate Deficiency
Jayatissa, Fernando and De Silva, 2017 ^A^ [16]	25 districts	Pregnant women	3.4 ^a^	NA	NA	NA
Wickramasinghe et al., 2017 [9]	Colombo MC area	Infants aged 6–6.5 months	1.1 ^a^	NA	NA	NA
Marasinghe et al., 2015 [10]	Ragama MOH area	Pre-school children aged 2–5 years	38.2 ^b^	5 ^d^	NA	NA
de Lanerolle-Dias et al., 2012 [13]	Western Province	Girls who dropped out of school (age 15–19 years)	NA	NA	W-1.7 NW-1.8 ^f^	T-28 ^g^ (W-35.9, NW-24.7)
Hettiarachchi and Liyanage, 2012 [11]	Galle district	Pre-school children aged 3–5 years	5 ^c^	>25 ^e^	NA	M 41, F 32 ^h^
Thoradeniya et al., 2006 [14]	Colombo MC area	Adolescent girls and young women	NA	NA	0·44 ^f^	43.6 ^h^
Hettiarachchi et al., 2006 [12]	Galle district	Secondary school children aged 12–16 years	NA	NA	NA	53.3 ^h^ (M 54.6, F 52.5)
Jayatissa and Gunathilaka, 2006 ^A^ [18]	20 districts	Children aged 6–60 months	29.3 ^a^	NA	NA	NA
		Non-pregnant women aged 15–49 years	14.9 ^a^	NA	NA	NA

NA, not available. M, Male; F, Female; MC, Municipal council; MOH, Medical officer of health; T, Total population; W, working adolescent girls who had dropped out of school; NW, not working adolescent girls who had dropped out of school. ^A^ National survey. Vitamin A deficiency in the different studies was defined using the following criteria: (a) Serum retinol < 20 μg/dL; (b) serum vitamin A < 20 μg/dL; and (c) serum retinol < 0.70 μmol/L. Vitamin D deficiency was defined as serum 25-hydroxyvitamin D: (d) <10 ng/mL; and (e) <35 nmol/L. (f) Vitamin B_12_ deficiency was defined as serum vitamin B_12_ < 150 pg/mL. Folate deficiency was defined as: (g) serum folic acid < 3 μg/L; and (h) serum folate < 3.00 ng/L.

**Table 9:** Prevalence and risk of anemic subject having MNDs.

Study	Population Studied	Micronutrient status	Prevalence (%)	*p* Value #	Risk *	*p* Value #
Thoradeniya et al., 2006 [14] ^A^	Adolescent girls and young women in Colombo	Serum folic acid < 3 ng/mL	62·1	0·001	NA	NA
		SF < 12 mg/L	65·2	< 0·001	NA	NA
		SF < 20 mg/L	74·2	< 0·001	NA	NA
		Serum folic acid < 3 ng/mL and SF < 12 mg/L	43·9	< 0·001	NA	NA
		Serum folic acid < 3 ng/mL and SF <20 mg/L	51·5	< 0·001	NA	NA
Hettiarachchi and Liyanage, 2012 [11]	Preschool children in Galle district	Serum folate < 3 ng/L	NA	NA	M 2.2 (1.8,6.0) F 1.5 (1.1, 4.3)	0.02
		SF 12.00 mg/L	NA	NA	F 2.4 (1.0,5.5)	0.03
		Serum calcium 1.20 mmol/L	NA	NA	F 2.4 (1.2, 15.3)	0.001
		Serum retinol < 0.70 mmol/L	NA	NA	F 3.8 (1.5, 9.2)	0.003
		Iron and folate deficiency	NA	NA	F 2.3 (1.8, 7.5)	0.02
		Folate deficiency and serum zinc < 9.945 mmol/L	NA	NA	F 4.6 (1.1, 20.9)	0.03
Hettiarachchi et al., 2006 [12]	Secondary school children in Galle district	SF < 30 μg/L	M 30.2 F 47.8	NA	F 1.58 (1.11, 2.23)	0.01
		SF < 30 μg/L and serum zinc < 9.95 μmol/L	NA	NA	F 0.64 (0.43,0.96)	0.029
Marasinghe et al., 2015 [10]	Preschool children in Ragama MOH area	Serum vitamin A < 20 μg/dL	60	NA	NA	NA
		Serum zinc < 9.9 μmol/L	60	NA	NA	NA
		Serum vitamin D < 10 ng/mL	56	NA	NA	NA
Jayatissa et al., 2014 [17]	Children aged 6–59 months	SF < 30 μg/dL	52.3	NA	NA	NA
		Serum zinc < 65 μg/dL	6.7	NA	NA	NA

* Age adjusted odds ratios, 95% confidence interval in parentheses. # *p* values less than 0.05 were considered significant. ^A^ Chi-square test was used to compare the iron and folic acid status in anemic and non-anemic subjects. NA, not available. M, Male; F, Female.
